# Supply Chain and Workforce Capacity Development for Diagnostic Services in Low-and Lower Middle-Income Sub-Saharan Countries

**DOI:** 10.5334/aogh.5423

**Published:** 2026-11-03

**Authors:** Maarten C. Bosland, Modupe Kuti, Becaye Fall, Emilia Virginia Noormahomed, Coumba Toure Kane

**Affiliations:** 1 Department of Pathology and Center for Global Health, College of Medicine, University of Illinois Chicago, Chicago, USA; 2 Department of Chemical Pathology, College of Medicine, University of Ibadan, Ibadan, Nigeria; 3 Ministry of Health and Public Hygiene, Dakar, Sénégal; 4 Department of Microbiology, Faculty of Medicine, Universidade de Eduardo Mondlane, Maputo, Mozambique; 5 Mozambique Institute for Health Education and Research-MIHER, Maputo, Mozambique; 6 University of California San Diego, San Diego, USA; 7 Department of Applied Biological and Pharmaceutical Sciences, Bacteriology and Virology, Faculty of Medicine Pharmacy and Odontology, Cheikh Anta Diop University, Dakar, Sénégal

**Keywords:** supply chains, workforce development, resource limitations, pathology, laboratory medicine, diagnostic imaging, Sub-Saharan Africa

## Abstract

The diagnosis of communicable and noncommunicable diseases cannot be made without radiology/imaging and pathology and laboratory medicine (PALM) services. These diagnostic services are often an under-recognized vital component of health care systems. Supply chain challenges and limited human resource capacity and capability are major impediments to delivering accurate, timely, and comprehensive diagnostic services in low-income and lower-middle-income countries (LMICs) in sub-Saharan Africa (SSA) and other low-resource regions. This viewpoint provides a current assessment of these supply chain and human resource challenges and addresses possible solutions. Chronic underfunding, lack of transparency, and an over-dependence on inadequate or obsolete methods of supply chain management at all levels are common in many low-resource regions. Furthermore, overdependence on high-income countries for basic diagnostic supplies jeopardizes diagnostic and health care services. Resource limitations impede attaining laboratory accreditation and certification. The scarcity of training programs at all levels of laboratory medicine and pathology as well as imaging and the serious problem of brain drain to high-income countries are major problems in many, if not all LMICs. Training in supply chain management is generally vastly underdeveloped in many low-resource countries. Diagnostic services in rural and remote low-resource settings are often very limited. Stakeholder engagement and the adoption of needs-based solutions to gaps in supply chain management and human resource capacity are essential, as well as political will, public–private partnerships, and adequate funding. Development of effective, sustainable local and regional policies and governance is essential to strengthen diagnostic supply chains and workforce training in SSA and other LMIC areas. Diagnostic services in rural and remote areas in many LMICs need to be vastly improved.

## Introduction and Background

Laboratory services still remain an under-recognized vital component of health care systems. The impact of these services goes unseen for the vast majority of patients, yet laboratories are the backbone of any health system. It is difficult to deliver effective and high-quality care to patients without knowing their exact diagnosis [1]. The diagnosis of epidemiologically significant communicable and noncommunicable diseases (NCDs) cannot be made without pathology and laboratory medicine services. Most clinical decisions in general hospital services are guided by laboratory and imaging findings, providing the *right* diagnosis, for the *right* patient, at the *right* time. Pathology and laboratory medicine (PALM) are important for an estimated 60-80% of diagnoses [2, 3], although this estimate is not well established and likely changes over time [4]. Radiology imaging and clinical and other observations add to PALM. In addition, these diagnostic services are also essential for disease staging and prognosis, ongoing assessment of clinical care, monitoring clinical response to treatment, disease surveillance and early detection of disease outbreaks, disease registries, and quality assurance within the health care system [5]. Laboratories are also major revenue providers for hospitals, making them both a scientific-medical and an economic pillar. They also generate the data that policymakers rely on for planning and interventions.

The provision of PALM services faces unique challenges in low-income countries and lower-middle-income countries (LMICs). The challenges are well known and include fragmented governance, limited financing, and high dependency on external donors. Performance gaps in health supply chains in LMICs significantly contribute to the inefficiency and underperformance of their pathology and laboratory health systems. In addition, lack of human resources, weak performance monitoring structures for supply chains, inadequate initial and continuous training programs, lack of transparency, over-reliance on outdated methods, such as manual data collection and record-keeping, and lack of monitoring and evaluation are also key factors in these countries. Equipment is often heterogeneous, poorly maintained, and unevenly distributed. Rural and peripheral laboratories are under-resourced and suffer from poor transportation and information technology infrastructures. In addition, unregulated private labs and weak compliance control further reduce the quality and comparability of results. In addition, these challenges weaken outbreak preparedness and reduce public trust in health systems. Assessing the maturity of laboratory systems is essential for identifying gaps and tracking progress toward key global health goals, including the targets set by the World Health Organization (WHO) International Health Regulations (IHR) and Universal Health Coverage (UHC) [6, 7], WHO World Health Assembly resolution WHA76.5 [8], and the WHO Global Diagnostics Coalition [9]. It is also critical for advancing equitable access to health care, integrating diagnostic services, and strengthening overall health system performance. The uninterrupted availability of health commodities depends on well-functioning, robust supply chain systems.

Thus, supply chain challenges and limited human resource capacity and capability in LMICs are major impediments to delivering accurate, timely, and comprehensive diagnostic services. These issues have long been recognized and addressed in some major publications in the past [10–13], including from the Lancet Commission on diagnostics [14, 15]. However, there are only a few more recent publications that are devoted to these challenges, particularly those regarding supply chain problems and human resource limitations [16, 17]. And regional efforts in sub-Saharan Africa (SSA) in addressing gaps in diagnostics supply chains and workforce development are mostly incipient [15, 18, 19]. Important issues of diagnostic network optimization, data systems, and costs and pricing of diagnostic services in LMICs are discussed in detail in a recent paper [18] and in a publication from the Africa Centres for Disease Control and Prevention (CDC) [19], but do not specifically address many supply chain and workforce development challenges.

The objective of this viewpoint is to re-energize attention on the critical role of supply chains and human resources for PALM services and diagnostic imaging in LMICs with a focus on SSA. The paper is based on presentations by the authors at the 2025 AFREhealth Symposium in Dakar, Senegal [20].

## Supply Chain Issues

All PALM and imaging modalities critically depend on an adequate, sustainable, and reliable supply chain for reagents and other consumables as well as equipment and spare parts. An often underappreciated but also critically important form of supply chain is that of timely and reliable equipment maintenance and repair [21].

Disruptions in the laboratory medicine and imaging supply chains usually appear in one of two forms: overt disruptions or covert disruptions. Overt problems are easy to recognize. These occur when the laboratory simply cannot provide a specific service or any service at all. This might be due to stockouts of essential reagents, expiration of reagents, or reagents damaged by poor storage or transport conditions. It can also happen when equipment breaks down, and spare parts or technical support are unavailable, or service contracts are not in place. Inadequate physical plant and local infrastructure for laboratory and storage space, electricity, water, internet, and safety, including radiation safety, also impact supply chains as do poor road conditions, limitation in air transportation, seasonal problems, particularly impacting rural regions. The need for cold chain conditions for an increasing range of diagnostic reagents poses additional complex challenges and risks of loss during transportation and because of inadequate storage. For imaging services involving X-rays, timely licensing and inspections can pose limitations, in addition to a lack of regular equipment maintenance.

Covert problems, sometimes called hidden or occult problems, are trickier. Here, the laboratory continues to provide services, but at the cost of enormous waste in the background. For example, large deliveries may arrive with very short shelf lives, leading to spoilage. Another source of waste is when multiple donors supply reagents or consumables to the same lab without coordination. For example, when donor A supports child health care, donor B supports HIV/AIDS services, and donor C supports maternal care, each donor may buy different reagents for different analytical instruments. The result is inefficiency and duplication. Another form of waste occurs when reagents are delivered before the equipment for which they are needed arrives, or before staff are trained to use them or the equipment. Unfortunately, this is not uncommon, even in recent times, despite available guidance from the WHO on how procurement practices should be coordinated [22].

Less often discussed, but very significant bottlenecks in supply chain management are associated with public procurement regulations. These are the legal requirements that dictate the policies, processes, and procedures by which government purchases are approved and paid for. While these rules are intended to streamline government purchasing, in practice they often do the opposite. Vendors supplying governments frequently encounter bureaucratic delays in approvals and payments, lack of transparency that enables corruption, and unpredictable payment timelines, making effective business planning difficult if not impossible [18]. In response, vendors typically raise their prices to protect themselves, ultimately increasing the cost of laboratory services further consuming scarce resources.

In short, poor supply chain management does not just deny patients essential services, it also squanders scarce and valuable resources. Yet, in many LMICs, structures for monitoring supply chain performance are weak, and no national logistics management databases exist. Local data gathering and record-keeping often occur manually, and quality management is suboptimal at best. Human resources in supply management are often limited by a lack of training and continuing education opportunities for supply chain staff. Compounding these problems, there is often a lack of standardization of laboratory equipment and techniques across local, regional, and national diagnostic services. Overall, chronic underfunding, lack of transparency, and an over-dependence on inadequate or obsolete methods of supply chain management at all levels is common in many low-resource regions. Furthermore, overdependence on high-income countries for some of the basic supplies will continue to jeopardize diagnostic and health care services.

## Human Resources Capacity Limitations

Adequate diagnostic services critically depend on the availability and effort of well-trained and supported human resources, at all levels. These human resources range from laboratory medicine scientists and physicians, pathologists, and radiologists to laboratory and radiology technicians and other technical support staff, such as information technologists, to procurement and inventory staff and physical plant and maintenance staff. Across most LMICs, there are serious limitations in human resources capacity at all these levels. Staff may not be well-oriented in procurement and supplies management, and training programs to address the unique issues of pathology, laboratory medicine, and imaging are scarce.

In-service and pre-service training programs for diagnostics technical staff are often weak in resource-limited countries, and the number of laboratory medicine scientists and physicians, pathologists, and radiologists is often vastly insufficient. Short staffing has negative impacts on quality assurance and on effective training of additional diagnostics staff.

Strengthening Laboratory Management Towards Accreditation (SLMTA) training programs are available for laboratory and diagnostic staff [23]. However, staff shortages frequently impede working toward accreditation, compounded by the paucity of training programs at all levels of laboratory medicine and pathology as well as imaging in many countries and the serious problem of brain drain to high-income countries. And training in supply chain management is generally vastly underdeveloped in many low-resource countries.

## Possible Solutions

To address the various gaps in supply chain management and human resource capacity, stakeholder engagement and the adoption of needs-based solutions are essential, which require political will. Adequate funding is also essential, recognizing that diagnostic services can not only generate revenue to cover their own costs, but also generate surplus revenue to cover other hospital needs and improve funds utilization through appropriate and timely evidence-based medical treatments. Avoiding unnecessary hospitalization and reducing antibiotic resistance are just some examples of the positive outcomes that can result from well-placed investment and improvement of laboratory services.

Strengthening health systems needs to involve the recognition that diagnostics are a critical component, not an afterthought. It requires integrating laboratories into surveillance networks, ensuring interoperability of digital systems, and securing supply chains for reagents and consumables [19]. It also requires investing in training and retaining laboratory professionals. If laboratories are embedded into the core of health planning, the entire system becomes more resilient to both infectious and NCDs. Remote evaluation of test results and especially radiology and histopathology images by medical specialists offers a promising approach to providing diagnostic services to distant rural hospitals and clinics, provided that they have functional quality-assured PALM and radiology capabilities.

For all of this to happen, national-level policy reform is essential. National Laboratory Directorates must be given a stronger legal status and a broader mandate to coordinate interventions, harmonize equipment, and attract skilled professionals. With stronger governance, data quality can be improved, equitable distribution of resources ensured, and laboratory systems aligned with national strategies such as UHC and emergency preparedness. This also makes it easier to engage with donors and regional partners in a coordinated way.

Central to solutions to supply chain and workforce development challenges is the availability of rigorous regional data on laboratory systems and diagnostic networks capacity as laid out by the Africa CDC in its geospatial diagnostics mapping project [18]. Country-by-country surveys of availability of PALM services have been conducted in several SSA countries [24–28].

### Supply chain issues

Policies to create and support an effective supply chain must recognize their considerable and challenging complexity. Elements assuring effective supply chain management include integration, operation, purchasing, distribution, agility, innovation, performance measurement, alignment, technology, and coordination. Components that ensure a seamless and efficient supply chain process and optimize resource use and customer satisfaction are many: procurement, inventory management, logistics, warehousing, order fulfillment, information technology, supplier relationship management, and demand forecasting. To make all this work well, adequate short- and long-term planning is essential, requiring effective policies and local institutional and national-level government support.

Procurement policies must recognize that laboratory supplies are not all the same, falling into four broad categories. The first category is that of one-off purchases such as purchases of equipment as well as investments in infrastructure upgrades which tend to be long-term and non-recurrent. The second group is the variable purchases of reagents and consumables, the quantities of which should be based on reliable, month-to-month consumption data. Purchases in this group are recurrent and predictable. A third group includes the fixed but recurrent purchases of items such as internal and external quality control materials, manufacturer-recommended preventive maintenance, and calibration of measurement tools and equipment. The fourth group is that of unpredictable purchases, typically for unanticipated breakdowns and emergency maintenance or for unexpected loss of reagents or consumables. Government policies on regulation must allow for access to resources for items in the above-listed groups. Clearly, logistics management information systems are essential for proper planning. The systems adopted should promote accountability, transparency, and sustainability, and platforms should be appropriate for various levels of users.

National and regional back-up systems of supplies that can be provided on short notice would go a long way to prevent local stockouts. Such an approach would necessitate strong support for national governments and regional entities such as ECOWAS, EAC, SDAC, and ECCAS. Prerequisites of this approach are standardization of diagnostic platforms and well-developed transportation within countries and across regions, including maintaining cold chain conditions. Few publications have addressed transportation supply chain issues, but other transportation approaches, such as for laboratory sample transportation in rural regions, may provide good models [29]. It will be critical to avoid customs and other barriers at national borders and delays and added costs associated with middlemen and national procurement systems.

A similar approach is conceivable for adequate and timely equipment repair and maintenance, which is critical for not only PALM services but also radiology and ultrasound imaging. A small number of highly trained and experienced technicians could be located centrally to either travel to laboratories in need of their services or provide remote support to local technicians. This would also require strong national and regional-level support, including funding and facilities. In addition, ongoing and sustainable interactions between national/regional technical support staff and local technical staff will be essential to make such an approach work. For imaging involving X-rays, national and regional-level support for equipment inspections and licensing is critically important in addition to equipment repair and maintenance.

The vast majority of diagnostic equipment and supplies used in LMICs is derived from manufacturers and suppliers in high-income countries. Public-private partnerships between suppliers and LMIC users have enormous potential in this regard and can be a game changer if properly regulated. For example, framework contracts and leasing models with suppliers can reduce costs and ensure maintenance of equipment. Private partners can bring innovation, logistics expertise, and digital solutions, while the public sector guarantees equity, regulation, and alignment with national priorities. Hybrid public-private partnerships laboratories in underserved regions with shared governance could also be explored. Importantly, public-private partnerships must be structured to avoid fragmentation and ensure that public health goals come first.

Local and NGO investment in local, often small-scale, manufacturing companies in LMICs has the potential to provide a sustainable supply chain for some essential diagnostics. Examples of this could be the development of SSA vaccine producers [30, 31]. Public-private partnerships can provide considerable support for such local suppliers. LMIC manufacturers can also produce diagnostics and platforms that are not of interest for high-income-based companies but highly appropriate for many low-resource contexts. This has enormous potential to lead to sustainable regional and LMIC context-appropriate supply chains.

### Human resources development

Diagnostic workforce development not only needs to focus on acquiring core technical skills, but also foster values like humility and teamwork, and adapt to new technologies and patient-centered approaches. Developing competency in diagnostic testing should be a central goal in pursuing laboratory accreditation. Digital tools, remote learning, and AI can be leveraged as powerful approaches to training, improving communication, and addressing health disparities through strategies such as community-based training and e-learning; the ECHO platform is a good example [32].

Implementation of effective programs of continuing education on diagnostic services and laboratory management is critical for developing a sustainable and high-quality workforce. Collaboration with various organizations engaged in medical laboratory education is important for the purpose of improving the quality of training. Of note, training modules and programs must be flexible enough to accommodate the schedules of often busy noncore supply chain practitioners. Regional harmonization of curricula for training PALM and imaging technicians and accreditation of training programs could augment their effective implementation. Similarly, harmonization of residency and fellowship programs across regions would strengthen training of PALM scientists and physicians as well as radiologists.

With the increasing emergence of NCDs, health workers need to be provided with training on NCD-specific guidelines and diagnostic procedures to improve service quality.

### Policies and health systems strengthening

Development of effective and sustainable policies is essential to strengthen diagnostic supply chains and diagnostic workforce training. Systems for collecting, monitoring, and analyzing data on diagnostic services and how they support treatment and care outcomes, necessary for evidence-based decision-making and tracking progress, need to be strengthened in many low-resource environments. And supply chains and workforce development for diagnostic services need to be part of any health systems strengthening efforts.

Improving supply chains and workforce training for diagnostic services for infectious and NCDs requires a multi-faceted approach, including strengthening of primary health care; integrating services to address co-occurring conditions; leveraging digital solutions for wider access and efficiency of diagnostic services; and improving diagnostic infrastructure and resource availability (such as diagnostic equipment, effective cold chains, and adequate transportation and storage conditions). Strategies need to increasingly involve training of diagnostic workers on NCDs, which need to be aligned with the implementation of integrated national strategies for NCD care, prevention and control.

Diagnostic services need to be improved in rural and remote low-resource settings in SSA. Decentralization is crucial for this. Mobile sample and supply chain transport systems, digital platforms for remote result reporting and data collection, and point-of-care testing, telepathology, and teleradiology can make diagnostics more accessible in the periphery. Policies should ensure that international funding also supports peripheral laboratories. And by developing public-private partnerships with local peripheral services, diagnostic coverage can be sustainably extended. Ultimately, equity in diagnostics is essential for equity in health.

## Concluding Remarks

A key message for the future of diagnostic services in SSA and other limited or low-resource regions around the globe is that pathology and medical laboratories and imaging resources are not just support services; they are central to health security, clinical care, and even economic sustainability. Strengthening governance, investing in effective supply chains, workforce, and diagnostic infrastructure (including telepathology and teleradiology), and leveraging regional and public-private partnerships will allow LMICs to build laboratory networks that are resilient, comparable across countries, and prepared for future health threats. The future of diagnostic services lies in increased use of digitalization and data collection and monitoring, sustainable financing, in-country and regional manufacturing of diagnostics, and regional cooperation as essential components of health system strengthening and UHC.
